# Ethyl 5-amino-3-methyl­sulfan­yl-1*H*-pyrazole-4-carboxyl­ate

**DOI:** 10.1107/S1600536808035095

**Published:** 2008-11-08

**Authors:** Yan Li, Teng-Fei Shao, Jian-Wu Wang

**Affiliations:** a School of Chemistry and Chemical Engineering, Shandong University, Jinan 250100, People’s Republic of China

## Abstract

In the title compound, C_7_H_11_N_3_O_2_S, bond lengths and angles are within normal ranges. The crystal packing is stabilized by inter­molecular N—H⋯O hydrogen bonds, linking the mol­ecules into infinite one-dimensional chains along the *a* axis.

## Related literature

For the biological activity, see: Hanefeld *et al.* (1996 ▶). For a similar structure, see: Ren *et al.* (2004 ▶).
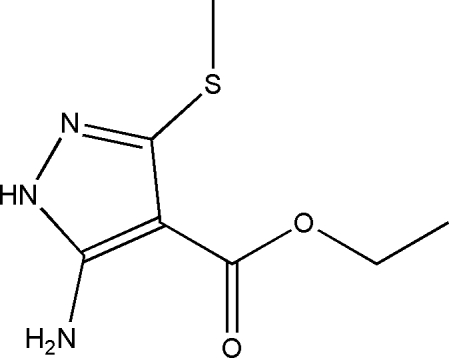


## Experimental

### 

#### Crystal data

C_7_H_11_N_3_O_2_S
*M* *_r_* = 201.25
Triclinic, 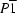

*a* = 7.0012 (7) Å
*b* = 7.5870 (8) Å
*c* = 10.1055 (10) Å
α = 81.038 (2)°
β = 72.173 (2)°
γ = 65.643 (1)°
*V* = 465.26 (8) Å^3^
*Z* = 2
Mo *K*α radiation
μ = 0.32 mm^−1^
*T* = 273 (2) K
0.10 × 0.10 × 0.05 mm

#### Data collection

Bruker SMART CCD area-detector diffractometer
Absorption correction: multi-scan (*SADABS*; Sheldrick, 1996 ▶) *T*_min_ = 0.969, *T*_max_ = 0.984
2317 measured reflections
1624 independent reflections
1488 reflections with *I* > 2σ(*I*)
*R*_int_ = 0.013

#### Refinement

*R*[*F* ^2^ > 2σ(*F* ^2^)] = 0.044
*wR*(*F* ^2^) = 0.127
*S* = 1.08
1624 reflections
118 parameters
H-atom parameters constrained
Δρ_max_ = 0.45 e Å^−3^
Δρ_min_ = −0.24 e Å^−3^

### 

Data collection: *SMART* (Bruker, 2001 ▶); cell refinement: *SAINT* (Bruker, 2001 ▶); data reduction: *SAINT*; program(s) used to solve structure: *SHELXTL* (Sheldrick, 2008 ▶); program(s) used to refine structure: *SHELXTL*; molecular graphics: *SHELXTL*; software used to prepare material for publication: *SHELXTL*.

## Supplementary Material

Crystal structure: contains datablocks I, global. DOI: 10.1107/S1600536808035095/hg2437sup1.cif

Structure factors: contains datablocks I. DOI: 10.1107/S1600536808035095/hg2437Isup2.hkl

Additional supplementary materials:  crystallographic information; 3D view; checkCIF report

## Figures and Tables

**Table 1:** Hydrogen-bond geometry (Å, °)

*D*—H⋯*A*	*D*—H	H⋯*A*	*D*⋯*A*	*D*—H⋯*A*
N1—H1*D*⋯O2^i^	0.86	2.16	2.914 (3)	146
N2—H2*C*⋯O2^i^	0.86	2.34	3.019 (3)	137

Symmetry code: (i) 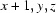.

**Fig. 1.**
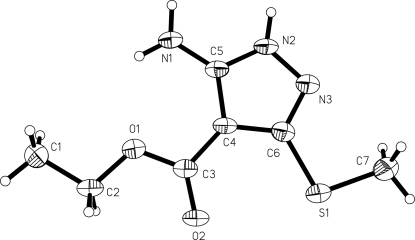
View of the title compound (I), with displacement ellipsoids drawn at the 40% probability level.

## supplementary crystallographic information

### Comment

The title compound, is an important intermediate in synthesis of heterocyclic compounds (Hanfeld *et al.*, 1996) in particular, in producing imidazo[1,2-*b*]pyrazole derivatives. Here, we report the crystal structure of (I).

In compound (I), all bond lengths and angles are normal and in a good agreement with those reported previously (Ren *et al.*, 2004). The pyrazole ring C4/C5/C5/N2/N3 and bonded atoms N1, S1, C3, O1, C2 and C1 are coplanar, the largest deviation from the mean plane being 0.053 (2)Å for atom C3. The crystal packing is stabilized by intermolecular N—H···O hydrogen bonds, linking the molecules into infinite one-dimensional chain along the *a* axis.

### Experimental

A round-bottomed flask fitted with a dropping funnel was charged with 11.2 g (0.2 mol) potassium hydroxide in 200 ml MeCN. The solution was cooled in an ice bath. Through the dropping funnel 22.7 g (0.2 mol) ethyl cyanoacetate was added gradually. After stirring at 273K for 0.5 h, 15.2 g(0.2 mol) carbon bisulfide was added while vigorous stirring. Keep stirring for 1 h, 50.4 g(0.4 mol) dimethyl sulfate was added through the drop funnel, then left overnight. The reaction mixture was filtered and filtrate evaporated on a rotary evaporator to remove the solvent. The mixture was dissolved in 50 ml ethanol, then through a drop funnel 12.5 g (0.2 mol) of hydrazine hydrate was added. The solution was evaporated *in vacuo* to afford crude product, which was purified by column chromatography to give the desired product 34.7 g, yield 86.3%. Crystals suitable for X-ray diffraction analysis were obtained by slow evaporation of a methanol solution at room temperature for one week.

### Refinement

All H atoms were placed in calculated positions, with C—H = 0.93 or 0.97 Å, N–H = 0.86 Å, and included in the final cycles of refinement using a riding model, with *U*_iso_(H) = 1.2 (1.5 times for methyl) times *U*_eq_(C,*N*).

### Crystal data

**Table:**

C_7_H_11_N_3_O_2_S	*Z* = 2
*M**_r_* = 201.25	*F*_000_ = 212
Triclinic, *P*1	*D*_x_ = 1.437 Mg m^−^^3^
Hall symbol: -P 1	Mo *K*α radiation λ = 0.71073 Å
*a* = 7.0012 (7) Å	Cell parameters from 1750 reflections
*b* = 7.5870 (8) Å	θ = 3.0–27.5º
*c* = 10.1055 (10) Å	µ = 0.32 mm^−^^1^
α = 81.038 (2)º	*T* = 273 (2) K
β = 72.173 (2)º	Block, yellow
γ = 65.6430 (10)º	0.10 × 0.10 × 0.05 mm
*V* = 465.26 (8) Å^3^	

### Data collection

**Table:**

Bruker SMART CCD area-detector diffractometer	1624 independent reflections
Radiation source: fine-focus sealed tube	1488 reflections with *I* > 2σ(*I*)
Monochromator: graphite	*R*_int_ = 0.013
*T* = 273(2) K	θ_max_ = 25.0º
φ and ω scans	θ_min_ = 2.1º
Absorption correction: multi-scan(SADABS; Sheldrick, 1996)	*h* = −8→8
*T*_min_ = 0.969, *T*_max_ = 0.984	*k* = −8→9
2317 measured reflections	*l* = −5→11

### Refinement

**Table:**

Refinement on *F*^2^	Secondary atom site location: difference Fourier map
Least-squares matrix: full	Hydrogen site location: inferred from neighbouring sites
*R*[*F*^2^ > 2σ(*F*^2^)] = 0.044	H-atom parameters constrained
*wR*(*F*^2^) = 0.127	*w* = 1/[σ^2^(*F*_o_^2^) + (0.0669*P*)^2^ + 0.2967*P*] where *P* = (*F*_o_^2^ + 2*F*_c_^2^)/3
*S* = 1.08	(Δ/σ)_max_ < 0.001
1624 reflections	Δρ_max_ = 0.45 e Å^−^^3^
118 parameters	Δρ_min_ = −0.24 e Å^−^^3^
Primary atom site location: structure-invariant direct methods	Extinction correction: none

### Special details

**Table:**

Geometry. All e.s.d.'s (except the e.s.d. in the dihedral angle between two l.s. planes) are estimated using the full covariance matrix. The cell e.s.d.'s are taken into account individually in the estimation of e.s.d.'s in distances, angles and torsion angles; correlations between e.s.d.'s in cell parameters are only used when they are defined by crystal symmetry. An approximate (isotropic) treatment of cell e.s.d.'s is used for estimating e.s.d.'s involving l.s. planes.
Refinement. Refinement of *F*^2^ against ALL reflections. The weighted *R*-factor *wR* and goodness of fit *S* are based on *F*^2^, conventional *R*-factors *R* are based on *F*, with *F* set to zero for negative *F*^2^. The threshold expression of *F*^2^ > σ(*F*^2^) is used only for calculating *R*-factors(gt) *etc*. and is not relevant to the choice of reflections for refinement. *R*-factors based on *F*^2^ are statistically about twice as large as those based on *F*, and *R*- factors based on ALL data will be even larger.

### Fractional atomic coordinates and isotropic or equivalent isotropic displacement parameters (Å^2^)

**Table:**

	*x*	*y*	*z*	*U*_iso_*/*U*_eq_	
S1	0.46271 (10)	0.72168 (10)	0.13781 (7)	0.0506 (3)	
O1	0.3262 (3)	0.7811 (3)	0.61242 (17)	0.0461 (4)	
O2	0.1914 (3)	0.7558 (3)	0.44317 (18)	0.0486 (5)	
N2	0.8938 (3)	0.7010 (3)	0.3056 (2)	0.0435 (5)	
H2C	1.0232	0.6889	0.3013	0.052*	
C3	0.3414 (4)	0.7574 (3)	0.4801 (2)	0.0383 (5)	
N1	0.7639 (3)	0.7355 (3)	0.5482 (2)	0.0510 (6)	
H1D	0.8881	0.7260	0.5535	0.061*	
H1E	0.6578	0.7515	0.6223	0.061*	
C5	0.7355 (3)	0.7256 (3)	0.4242 (2)	0.0368 (5)	
C2	0.1246 (4)	0.8045 (4)	0.7109 (3)	0.0418 (6)	
H2A	0.0903	0.6923	0.7138	0.050*	
H2B	0.0088	0.9182	0.6866	0.050*	
C6	0.6221 (4)	0.7165 (3)	0.2425 (3)	0.0387 (5)	
N3	0.8282 (3)	0.6970 (3)	0.1904 (2)	0.0450 (5)	
C4	0.5518 (3)	0.7352 (3)	0.3906 (2)	0.0360 (5)	
C1	0.1484 (5)	0.8274 (4)	0.8514 (3)	0.0542 (7)	
H1A	0.0146	0.8433	0.9215	0.081*	
H1B	0.1818	0.9392	0.8472	0.081*	
H1C	0.2638	0.7143	0.8740	0.081*	
C7	0.6528 (5)	0.7014 (5)	−0.0316 (3)	0.0626 (8)	
H7A	0.5843	0.7025	−0.1009	0.094*	
H7B	0.7777	0.5824	−0.0347	0.094*	
H7C	0.6977	0.8085	−0.0493	0.094*	

### Atomic displacement parameters (Å^2^)

**Table:**

	*U*^11^	*U*^22^	*U*^33^	*U*^12^	*U*^13^	*U*^23^
S1	0.0384 (4)	0.0712 (5)	0.0510 (4)	−0.0245 (3)	−0.0179 (3)	−0.0052 (3)
O1	0.0381 (9)	0.0649 (11)	0.0429 (9)	−0.0248 (8)	−0.0141 (7)	−0.0026 (8)
O2	0.0306 (9)	0.0707 (12)	0.0543 (11)	−0.0249 (8)	−0.0170 (8)	−0.0035 (9)
N2	0.0280 (9)	0.0626 (13)	0.0486 (12)	−0.0232 (9)	−0.0139 (8)	−0.0029 (9)
C3	0.0304 (11)	0.0412 (12)	0.0469 (13)	−0.0150 (9)	−0.0144 (10)	0.0001 (10)
N1	0.0336 (11)	0.0840 (16)	0.0477 (12)	−0.0301 (11)	−0.0154 (9)	−0.0060 (11)
C5	0.0286 (11)	0.0430 (12)	0.0450 (12)	−0.0174 (9)	−0.0143 (9)	−0.0002 (10)
C2	0.0311 (11)	0.0468 (13)	0.0547 (14)	−0.0174 (10)	−0.0189 (10)	−0.0003 (11)
C6	0.0324 (11)	0.0389 (12)	0.0460 (13)	−0.0151 (9)	−0.0100 (10)	−0.0022 (9)
N3	0.0335 (10)	0.0597 (13)	0.0463 (12)	−0.0218 (9)	−0.0105 (9)	−0.0043 (9)
C4	0.0254 (10)	0.0398 (11)	0.0475 (13)	−0.0141 (9)	−0.0141 (9)	−0.0016 (9)
C1	0.0513 (15)	0.0736 (18)	0.0446 (14)	−0.0320 (14)	−0.0093 (12)	−0.0064 (13)
C7	0.0547 (17)	0.095 (2)	0.0483 (16)	−0.0347 (16)	−0.0180 (13)	−0.0050 (15)

### Geometric parameters (Å, °)

**Table:**

S1—C6	1.744 (2)	C5—C4	1.399 (3)
S1—C7	1.801 (3)	C2—C1	1.522 (3)
O1—C3	1.343 (3)	C2—H2A	0.9700
O1—C2	1.415 (3)	C2—H2B	0.9700
O2—C3	1.222 (3)	C6—N3	1.328 (3)
N2—C5	1.336 (3)	C6—C4	1.435 (3)
N2—N3	1.385 (3)	C1—H1A	0.9600
N2—H2C	0.8600	C1—H1B	0.9600
C3—C4	1.429 (3)	C1—H1C	0.9600
N1—C5	1.346 (3)	C7—H7A	0.9600
N1—H1D	0.8600	C7—H7B	0.9600
N1—H1E	0.8600	C7—H7C	0.9600
			
C6—S1—C7	100.66 (12)	N3—C6—C4	111.9 (2)
C3—O1—C2	116.79 (18)	N3—C6—S1	122.18 (19)
C5—N2—N3	113.11 (18)	C4—C6—S1	125.90 (17)
C5—N2—H2C	123.4	C6—N3—N2	104.02 (19)
N3—N2—H2C	123.4	C5—C4—C3	129.2 (2)
O2—C3—O1	123.1 (2)	C5—C4—C6	103.93 (19)
O2—C3—C4	125.2 (2)	C3—C4—C6	126.9 (2)
O1—C3—C4	111.67 (19)	C2—C1—H1A	109.5
C5—N1—H1D	120.0	C2—C1—H1B	109.5
C5—N1—H1E	120.0	H1A—C1—H1B	109.5
H1D—N1—H1E	120.0	C2—C1—H1C	109.5
N2—C5—N1	122.8 (2)	H1A—C1—H1C	109.5
N2—C5—C4	107.0 (2)	H1B—C1—H1C	109.5
N1—C5—C4	130.2 (2)	S1—C7—H7A	109.5
O1—C2—C1	106.85 (18)	S1—C7—H7B	109.5
O1—C2—H2A	110.4	H7A—C7—H7B	109.5
C1—C2—H2A	110.4	S1—C7—H7C	109.5
O1—C2—H2B	110.4	H7A—C7—H7C	109.5
C1—C2—H2B	110.4	H7B—C7—H7C	109.5
H2A—C2—H2B	108.6		
			
C2—O1—C3—O2	0.2 (3)	N1—C5—C4—C3	−0.4 (4)
C2—O1—C3—C4	−179.98 (19)	N2—C5—C4—C6	−0.8 (2)
N3—N2—C5—N1	−179.3 (2)	N1—C5—C4—C6	179.9 (2)
N3—N2—C5—C4	1.3 (3)	O2—C3—C4—C5	−176.5 (2)
C3—O1—C2—C1	179.7 (2)	O1—C3—C4—C5	3.6 (3)
C7—S1—C6—N3	−1.3 (2)	O2—C3—C4—C6	3.1 (4)
C7—S1—C6—C4	177.5 (2)	O1—C3—C4—C6	−176.7 (2)
C4—C6—N3—N2	0.6 (3)	N3—C6—C4—C5	0.1 (3)
S1—C6—N3—N2	179.63 (16)	S1—C6—C4—C5	−178.88 (17)
C5—N2—N3—C6	−1.2 (3)	N3—C6—C4—C3	−179.6 (2)
N2—C5—C4—C3	178.9 (2)	S1—C6—C4—C3	1.4 (4)

### Hydrogen-bond geometry (Å, °)

**Table:**

*D*—H···*A*	*D*—H	H···*A*	*D*···*A*	*D*—H···*A*
N1—H1D···O2^i^	0.86	2.16	2.914 (3)	146
N2—H2C···O2^i^	0.86	2.34	3.019 (3)	137

Symmetry codes: (i) *x*+1, *y*, *z*.

## References

[bb1] Bruker (2001). *SMART* and *SAINT* Bruker AXS Inc., Madison, Wisconsin.

[bb2] Hanefeld, U., Rees, C. W. & White, A. J. P. (1996). *J. Chem. Soc., Perkin Trans. 1*, pp. 1545–1552.

[bb3] Ren, X. L., Wu, C., Hu, F. Z., Zou, X. M. & Yang, H. Z. (2004). *Chin. J. Chem. ***22**, 194–198.

[bb4] Sheldrick, G. M. (1996). *SADABS* University of Göttingen, Germany.

[bb5] Sheldrick, G. M. (2008). *Acta Cryst.* A**64**, 112–122. 10.1107/S0108767307043930 18156677

